# Supplementary material for: Store-Operated Ca2+ Entry in Skeletal Muscle Contributes to the Increase in Body Temperature during Exertional Stress
Source: Int J Mol Sci. 2022 Mar 29;23(7):3772. doi: 10.3390/ijms23073772 (PMC8998704; doi:10.3390/ijms23073772)
Supplement: Supplementary file 1 [file ijms-23-03772-s001.zip › ijms-1632662-supplementary.pdf]

International Journal of
Molecular Sciences

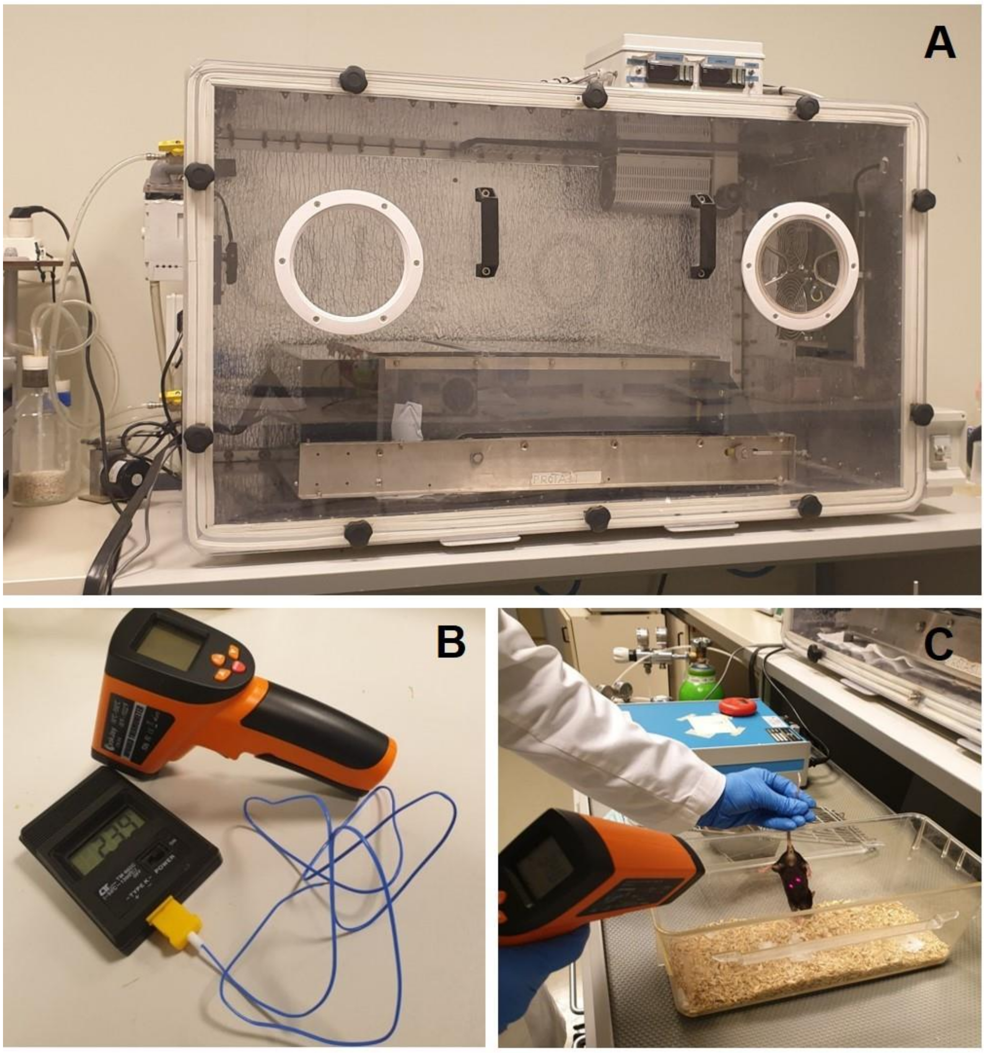

Figure S1. Environmental chamber and infrared thermometer. (**A**) The environmental chamber used during the ES protocol. B-C) Infrared thermometer (panel **B**) and its use during skin temperature measurement (panel **C**). See Suppl. Figure 2 for the outcome of these measurements.

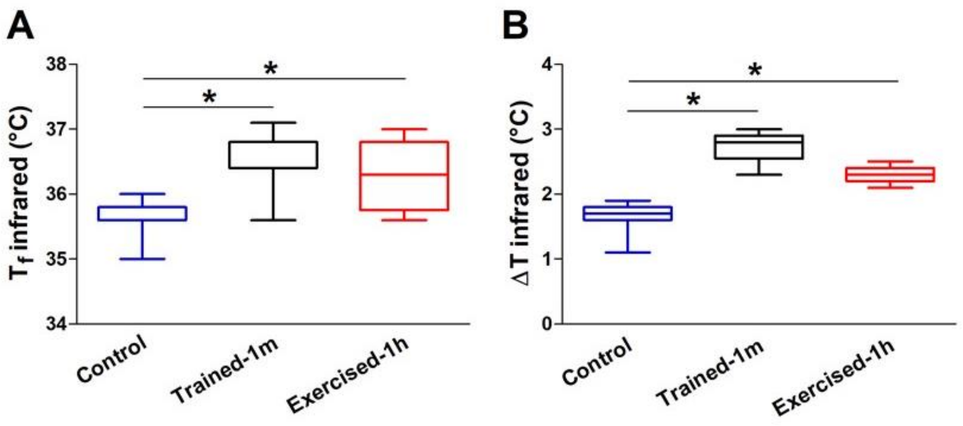

	Final temperature (T_f)	Delta temperature (ΔT)
Control	35.6 ± 0.3	1.6 ± 0.3
Trained-1m	36.5 ± 0.4*	2.7 ± 0.3*
Exercised-1h	36.3 ± 0.5*	2.3 ± 0.1*

Figure S2. Average skin temperature of mice following the ES protocol recorded with the infrared thermometer (as shown in Suppl. Fig. 1 B and C). A) Average cutaneous temperature recorded at the end of the ES protocol (T_f). B) Average changes in cutaneous temperature (ΔT) during ES protocol, i.e. the difference between temperatures measured at the beginning (T_0) and at the end (T_f) of the experiment. Data are shown as follow: i) the box extends from the 25th to 75th percentiles; the line in the middle of the box is plotted at the median and the whiskers go down to the smallest value and up to the largest (graphs in panels A and B); and ii) mean ± SEM (*p<0.05), as evaluated by two-tailed unpaired Student's t-test (Table). Sample size (number of tested mice): control = 8; trained-1m= 8; exercised-1h = 9.

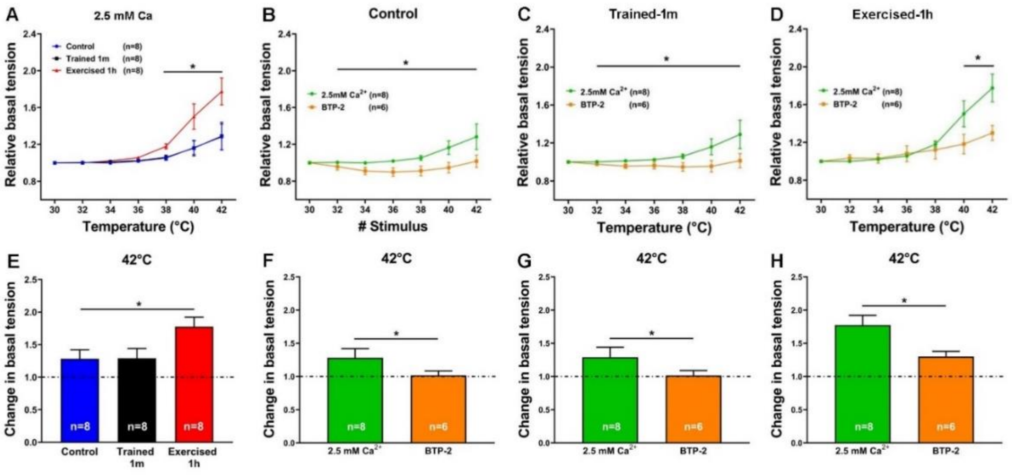

Figure S3. Relative and specific basal tension developed by isolated EDL muscles during an ex-vivo heat stress (HS) protocol. (**A–D**) Relative basal tension developed by EDL muscles during an ex-vivo HS protocol (i.e. 45-minute electrical stimulation with 60 single twitches, at 1 Hz, applied every 5 seconds). The chamber temperature was increased by 2°C (from 30°C to 42°C) every 5 minutes. Experiments were performed in presence of 2.5 mM of extracellular Ca^{2+} (panel A) or in a solution supplemented with 10 μM BTP-2 (panels B-D). (**E–H**) Specific basal tension recorded at 42°C, either in presence of 2.5 mM of extracellular Ca^{2+} (panel E) or in a solution supplemented with 10 μM BTP-2 (panels F-H). Data are shown as mean ± SEM; in panels (A,E): *p<0.05 = *indicate a difference between exercised-1h and both control and trained-

1m mice; in panels B-D and F-H: *$p<0.05$ = difference between 2.5mM Ca^{2+} and BTP-2; as evaluated by two-tailed unpaired Student's t-test). n = number of EDL muscles analysed.

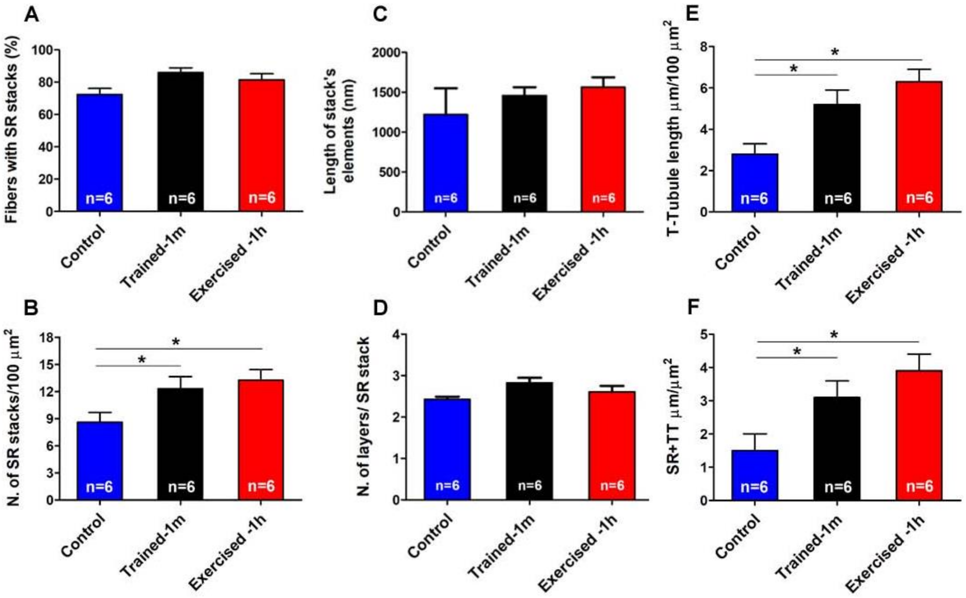

Figure S4. Quantitative EM analysis of SR-stacks and of T-tubule extension at the I band after the ES stress protocol**.** (**A** and **B**) Percentage of fibers containing SR-stacks and number of SR-stacks / 100 µm² of section. (**C** and **D**) Length of stack's elements (nm) and number of layers / SR-stack. (**E** and **F**) Extension of T-tubules at the I band in 100 µm² and extension of SR-TT contacts at the I band. Data are shown as mean ± SEM (*$p < 0.05$; the two ends of the line with asterisk indicate which two groups are being compared). n = number of EDL analysed.

Table S1. Average core temperature of mice following the ES protocol.

	**Final temperature (T_f)**	**Delta temperature (ΔT)**
**Control**	37.9 ± 0.2	1.7 ± 0.1
**Trained-1m**	38.9 ± 0.3*	2.7 ± 0.2*
**Exercised-1h**	38.8 ± 0.5*	2.3 ± 0.1*

Sample size (number of tested mice): control, n = 8; trained-1m, n = 8; exercised-1h, n = 9. Data are shown as mean ± SEM (*$p<0.05$, as evaluated by two-tailed unpaired Student's t-test).

Table S2. Quantitative analysis of structural damage in longitudinal sections of EDL fibers in basal condition.

	**% of fiber with no damage**	**% of altered fibers**
**Control**	98.6 ± 1.4	1.7 ± 1.4
**Trained-1m**	98.2 ± 1.8	2.4 ± 1.8
**Exercised-1h**	98.5 ± 1.5	2.1 ± 1.5

Sample size (number of tested mice): n= 3 for each sample group. Data are shown as mean ± SEM. Statistical significance was evaluated by two-tailed unpaired Student's t-test.

Table S3. Quantitative analysis of structural damage in longitudinal sections of EDL fibers, after the ES protocol.

	% of fiber with no damage	% of altered fibers
**Control**	94.0 ± 2.3	6.5 ± 2.3
**Trained-1m**	98.5 ± 0.9	1.9 ± 0.9*
**Exercised-1h**	93.1 ± 2.6	7.2 ± 2.6

Sample size (number of tested mice): n= 3 for each sample group. Data are shown as mean ± SEM (*$p < 0.05$ vs. control and vs. exercised-1h, as evaluated by two-tailed unpaired Student's t-test.).

Table S4. Quantitative EM analysis of mitochondria n./area and relative volume.

	No. of mitochondria /100 μm^2	Mitochondrial volume/total volume (%)
**Control**	26.8 ± 0.8	3.8 ± 0.1
**Trained-1m**	41.1 ± 1.2*	7.1 ± 0.2*
**Exercised-1h**	29.2 ± 0.9	4.0 ± 0.1

Sample size (number of tested mice): control, n = 6; trained-1m, n = 4; exercised-1h, n = 6. Data are shown as mean ± SEM (*$p < 0.05$ vs. control and vs. exercised-1h, as evaluated by two-tailed unpaired Student's t-test).
